# Oral adsorbent AST-120 ameliorates gut environment and protects against the progression of renal impairment in CKD rats

**DOI:** 10.1007/s10157-018-1577-z

**Published:** 2018-04-19

**Authors:** Ayumi Yoshifuji, Shu Wakino, Junichiro Irie, Ayumi Matsui, Kazuhiro Hasegawa, Hirobumi Tokuyama, Koichi Hayashi, Hiroshi Itoh

**Affiliations:** Department of Internal Medicine, School of Medicine, Keio University, 35 Shinanomachi, Shinjuku-ku, Tokyo, 160-8582 Japan

**Keywords:** CKD, Microbiota, Uremic toxin, Tight junction, Toll-like receptor

## Abstract

**Background:**

Oral charcoal adsorbent AST-120 (AST) is reported to ameliorate renal dysfunction by the absorption of toxic substance in the gut. Recent study revealed that, in CKD, gut environment is disturbed including the decrease in tight junctions and *Lactobacillus* (*Lact*). In this study, we examined whether AST improves the renal dysfunction through gut environment.

**Method:**

Six-week-old spontaneously hypertensive rats (SHR) were rendered CKD by 5/6th nephrectomy (Nx). SHRs were divided into SHR (Sham), SHR with Nx (Nx), and Nx given AST (Nx + AST) (*n* = 10, each). After 12 weeks, rats were killed and biochemical parameters were explored. The gut flora was analyzed. Furthermore, gut molecular changes in tight junctions and toll-like receptors were examined. We also investigated the effects of the combination therapy with AST and *Lact*.

**Results:**

The increase in serum urea nitrogen and urinary protein excretion in Nx was restored in Nx + AST. The increased renal glomerulosclerosis in Nx was ameliorated in Nx + AST. Increases in serum uremic toxins and IL-6 in Nx were ameliorated in Nx + AST. The gut flora analysis revealed that the decrease in *Lact* in Nx was restored in Nx + AST. The downregulation in the tight junction and TLR2 in Nx was mitigated by AST. However, combination therapy failed to exhibit additional effects.

**Conclusion:**

AST ameliorated renal function with the restoration of *Lact* and tight junction through TLR pathway, which would mitigate systemic inflammation and contributed to their renoprotective effects. Our study provides a novel mechanism of the renoprotective effects by AST.

## Introduction

The increase of chronic kidney disease (CKD) patients becomes the serious problem for humans because CKD elevates cardiovascular events and causes end-stage kidney disease [1]. Various treatments are used to delay the progression of CKD. However, only these treatments were not sufficient. As one of the pathogenic factors for progression, an unfavorable effect of uremic toxins recently attracts attention. Indoxyl sulfate (IS) and *p*-cresyl sulfate (PCS) are produced in the intestine by the bacteria from tryptophan or tyrosine, respectively, absorbed from the colon, metabolized into sulfur-conjugated substances, and excreted in the urine from the kidney [2]. Once accumulated by renal impairment, these substances cause the tissue damages through oxidative stress [3, 4]. Another uremic solute, indole acetic acid (IAA) is also shown to damage endothelial cells and increase cardiovascular events in CKD [5]. Charcoal adsorbent AST-120 (AST) is considered to be one of the options to delay the progression of CKD in Asia. It was designed to bind to uremic toxins in the colon and protect against the entry of IS precursors and reduced the plasma IS, thereby slowing the progression of CKD [6, 7]. Although the recent large clinical trial failed renal protective effects [8], several small sized clinical studies have shown its efficacy against the renal impairment [9, 10]. Besides, it was reported that the tight junction in the colon was disrupted in CKD, which was reversed by AST [11]. This effect is supposed to be beneficial to the maintenance of gut barrier function and inhibit the systemic inflammation evoked by the intrusion of various toxins from leaky gut [10]. In this way, the organ interrelationship between gut and kidney has been gaining the scientific interest, coined as ‘the intestinal-renal syndrome’ [12]. The modulation of this relationship can be a plausible strategy against CKD. We previously demonstrated that *Lactobacillus* (*Lact*) was decreased in CKD rats and that the supplementation of *Lact* improved the intestinal barrier, systemic inflammation and the kidney function [13]. However, the effects of AST on microbiota have not been reported thus far.

In this study, we demonstrated that AST elevates the population of *Lact* in CKD rats, which improved the disruption of intestinal barrier and systemic inflammation through TLR2 and TLR4. The present study provides the novel mechanism of AST through the modulation of gut environment.

## Materials and methods

### Animal experiments

SHR at 6 weeks of age (Charles river) were rendered CKD by 5/6th nephrectomy as described previously [14]. Rats were housed under controlled environmental conditions (12-h light–dark cycle) and were allowed free access to commercial food diet and water. Rats were randomly assigned to three experimental groups (*N* = 10 per group): (1) Sham, (2) Nx, (3) Nx + AST. In the second experiment, rats were randomly assigned to five experimental groups (*N* = 8 per group); (1) Sham, (2) Nx, (3) Nx + AST, (4) Nx + *Lact*, (5) Nx treated with AST and *Lact* (Nx + AST + *Lact*). *Lactobacillus acidophilus* is kindly provided from Nitto Pharmacy (Kyoto). AST-120 was kindly provided by Kureha Industry Co. (Tokyo). Twelve weeks after nephrectomy, body weight, systolic blood pressure (SBP) measured by tail-cuff method (KN-210, Natsume, Tokyo), and 24-h urinary protein excretion were evaluated. Afterwards, rats were killed and blood samples were collected after the overnight fasting. Plasma levels of blood urea nitrogen (BUN), creatinine, IS, PCS and IAA were measured as described previously [15, 16]. Tissue samples of kidney and ascending colon were removed and snap frozen. All experiments were performed in accordance with the animal experiment guideline of Keio University School of Medicine.

### Morphological examination

Kidneys were fixed in 10% formaldehyde and embedded in paraffin blocks. To evaluate the glomerular sclerosis and renal fibrosis, PAS-staining and Masson-trichrome staining were performed, respectively. Glomerulosclerosis is evaluated by counting sclerotic glomeruli and evaluated by glomerulosclerotic index [17, 18]. Fibrotic area was evaluated by measuring proportion of fibrotic area from 30 fields using Image-Pro Plus 3.0 (Media Cybernetics, Silver Spring, MD).

### The analysis of gut bacteria

Fecal samples were suspended in a solution containing 100 mM Tris–HCl (pH9.0) and 40 mM EDTA and were beaten at 5000 rpm for 3 min in the presence of glass beads (BioSpec Products). DNA was extracted using phenol–chloroform extraction, and the supernatant was subjected to isopropanol precipitation. Thereafter, the amplification of the fecal 16S rDNA, the restriction enzyme digestion, the size-fractionation, and the T-RFLP data analysis were conducted as previously reported [19]. PCR was performed as previously reported [13]. The T-RFLP patterns among samples were compared using the calculations of dissimilarity index [20]. The amounts of bacteria in each species in fecal samples were quantified by real-time quantitative PCR using the 7500 Fast Real-time PCR System (Applied Biosystems, USA) as previously reported [21]. All experiments were performed in duplicate and a melting curve analysis was done after amplification. The amounts of specific bacteria were calculated by the ratio to total bacteria.

### Immunoblotting

Ascending colon tissues were lysed and sonicated in lysis buffer and centrifuged at 15,000*g* for 15 min. Supernatant aliquots were subject to immunoblotting using primary antibody against Occludin, ZO-1, and Claudin-1 (Invitrogen). After blots were incubated with secondary antibody HRP-linked anti-rabbit IgG (GE healthcare, Backhamshire, England), immunoreactive bands were detected using an ECL detection kit (Amersham Biosciences, Uppsala, Sweden).

### Real-time polymerase chain reaction

Total RNA was extracted from ascending colon tissues using TRIzol reagent (Invitrogen). Equal amounts (1 µg) of total RNA from each sample were converted to cDNA by PrimeScript RT reagent Kit with gDNA Eraser (TaKaRa, Otsu, Japan) in a 20-µl reaction volume. Real-time PCR was performed for rat colon tissues, using an ABI Step One Plus sequence detector (PE Applied Biosystems, Tokyo, Japan). Levels of mRNA were normalized to those of β-actin. The primer sequences were shown in Table 1.

**Table 1:** Primer sequences for Real-time PCR

TLR2; sense 5′-GTACGCAGTGAGTGGTGCAAGT-3′
Antisense 5′-GGCCGCGTCATTGTTCTC-3′
TLR4; sense 5′-AATCCCTGCATAGAGGTACTTCC TAAT-3′
Antisense 5′-CTCAGATCTAGGTTCTTGGTTGAATAAG-3′
GAPDH; sense 5′-GTTACCAGGGCTGCCTCTC-3′
Antisense 5′-GGGTTTCCCGTTGATG ACC-3′

## Results

### AST attenuated renal damages in Nx

**Fig. 1:**
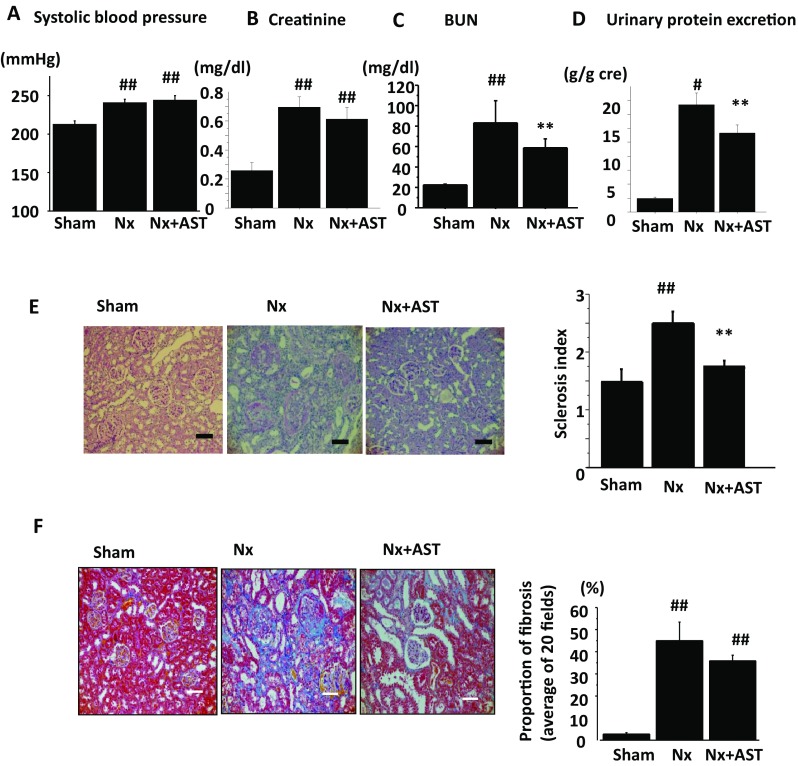
Effects of AST on renal impairment in nephrectomized (Nx) rats. Spontaneously hypertensive rats underwent sham operation (Sham), 5/6 nephrectomy (Nx) or Nx followed by AST treatment (Nx + AST), as described in “Materials and methods”. Measurements of SBP (**a**), serum creatinine level (**b**), blood urea nitrogen level (**c**), and daily urinary protein excretion (**d**). The evaluation of glomerular sclerosis by PAS staining (**e**) and interstitial fibrosis by Masson-trichrome staining (**f**) is shown. Left panels represent the quantification of glomerular sclerosis and interstitial fibrosis, and the right bar graph showed the quantification of stained areas. Scale bar, 100 µm. ^#^*P* < 0.05, ^##^*P* < 0.01 versus Sham; **P < 0.01 versus Nx. *N* = 10 per group

We investigated the effects of AST on the progression of CKD. Although chow intakes were not different among experimental groups, body weight decreased in Nx as compared with Sham, which were ameliorated in Nx + AST (Sham; 361.1 ± 8.8 g, Nx; 289.3 ± 26.8 g, Nx + AST; 320.0 ± 33.5 g). The increase in SBP in Nx was not ameliorated by AST (Fig. 1a). Although the increase in serum creatinine level in Nx was not altered in Nx + AST (Fig. 1b), the increase in BUN and urinary protein excretion in Nx was ameliorated in Nx + AST (Fig. 1c, d, respectively). Glomerulosclerosis was more evident in Nx as compared to those in sham, which was attenuated by the treatment with AST (Fig. 1e). The fibrotic area evaluated by Masson-trichrome staining was significantly increased in Nx as compared to that in Sham, although AST had no effects (Fig. 1f). These data supported the evidence for the renal protective effect by AST.

### AST reduced the serum levels of uremic toxins and inflammatory mediator

**Fig. 2:**
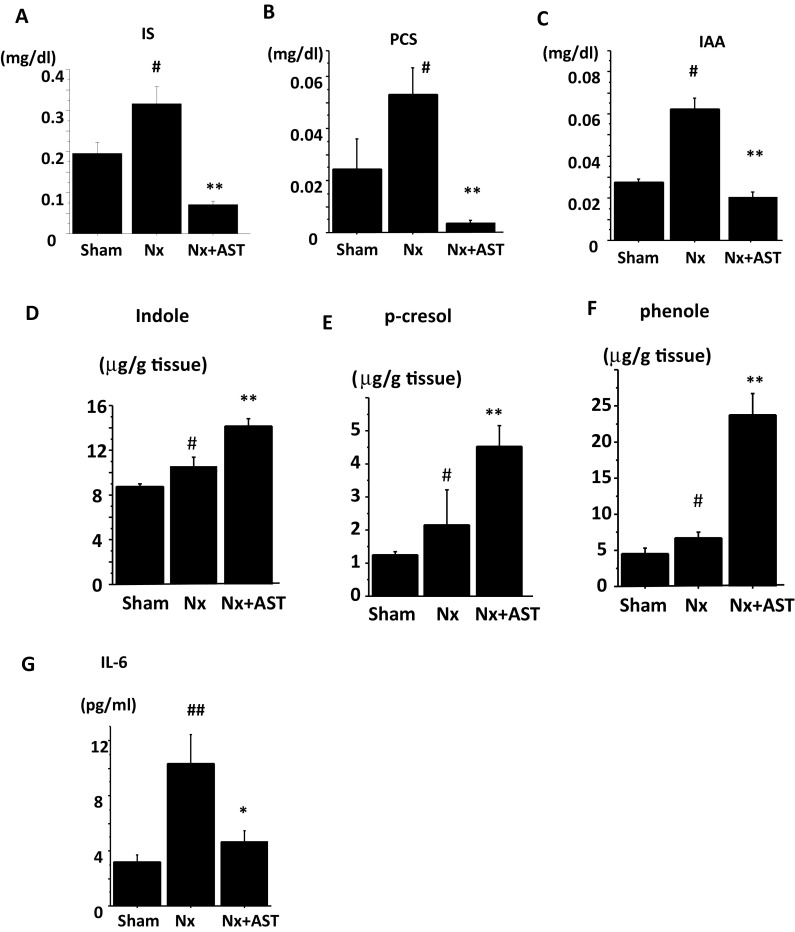
The effects by AST on uremic toxins and systemic inflammation in nephrectomized rats. Spontaneously hypertensive rats underwent sham operation (Sham), 5/6 nephrectomy (Nx) or Nx followed by AST treatment (Nx + AST), as described in “Materials and methods”. Serum concentrations of uremic toxins including indoxyl sulfate (IS, **a**), *p*-cresyl sulfate (PCS, **b**), and indole acetic acid (IAA, **c**) were compared among the groups. Fecal concentrations of the precursors of uremic toxins including indole (**d**), *p*-cresol (**e**), and phenol (**f**) were compared among the groups. Serum interleukin-6 (IL-6, **g**) was also shown. ^#^*P* < 0.05, ^##^*P* < 0.01 versus Sham; **P < 0.01 versus Nx. Sham: *N* = 6, Nx: *N* = 9, Nx + AST: *N* = 9

Main effects by AST were the absorbance of the uremic toxin precursors in the intestinal lumen and reduction of these plasma levels. Nx showed significant higher levels of serum IS, PCS, and IAA, and these levels were decreased in Nx + AST (Fig. 2a–c, respectively). Consistently, in Nx, fecal concentrations of indole, *p*-cresol and phenol were increased in Nx and these concentrations significantly elevated in Nx + AST, indicating that AST absorbed these substances in the intestine. Furthermore, the elevation of IL-6 in Nx was improved by AST (Fig. 2g), which implied that AST blocked the entry of bacterial toxins from gut, leading to the amelioration of systemic inflammation in Nx.

### AST restored the expressions of barrier molecules in the intestine

**Fig. 3:**
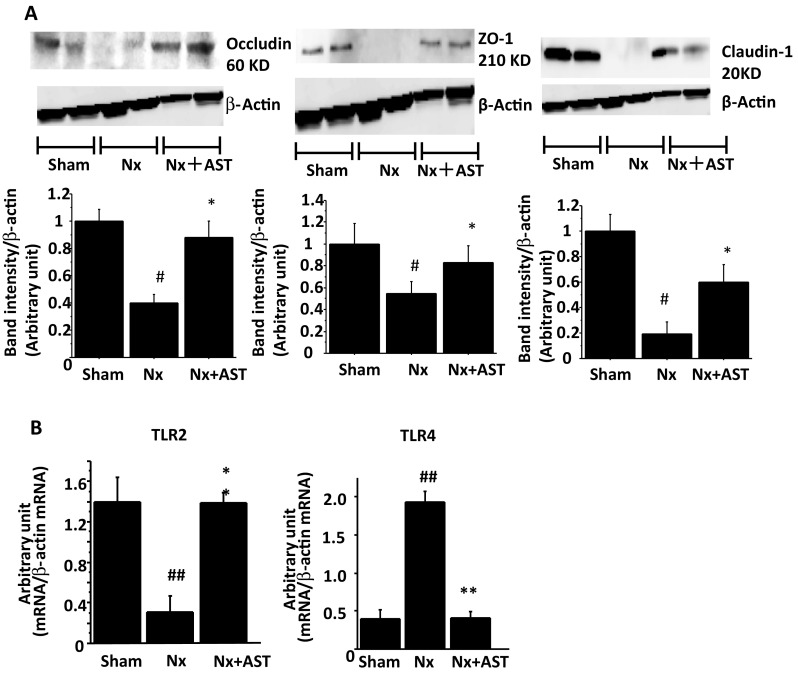
The effects of AST on intestinal environment in nephrectomized rats. Spontaneously hypertensive rats underwent sham nephrectomy (Sham), 5/6 nephrectomy (Nx), or Nx plus AST treatment (Nx + AST), as described in “Materials and methods”. **a** Expression levels of the tight junction proteins, occludin (left panel), ZO-1 (middle panel) and claudin-1 (right panel) in the colon were evaluated by immunoblotting. Each upper panel shows a representative immunoblot and each lower panel shows the densitometric analysis of each blot. **b** Levels of expressions of TLR2 (left) and TLR4 (right) in the colon were evaluated by RT-PCR. ^#^*P* < 0.05, ^##^*P* < 0.01 versus Sham; *P < 0.05, **P < 0.01 versus Nx. Sham: *N* = 7, Nx: *N* = 9, Nx + AST: *N* = 8

The reduction of the serum levels of colon bacteria-derived uremic toxins by AST implied that colon barrier would be improved by AST. The protein expressions of occludin, ZO-1 and claudin-1, key components of intestinal tight junction were all downregulated in Nx, which were mitigated by AST (Fig. 3a). To examine the mechanism to improve the tight junction, we evaluated toll-like receptors: TLR2 and TLR4 which are considered to be activated by intestinal microbiota in the intestine and to regulate the expressions of tight junction proteins [22, 23]. The decrease of TLR2 in Nx is mitigated by AST and the increase of TLR4 in Nx is downregulated by AST (Fig. 3b).

### AST restored *Lact* in Nx

**Fig. 4:**
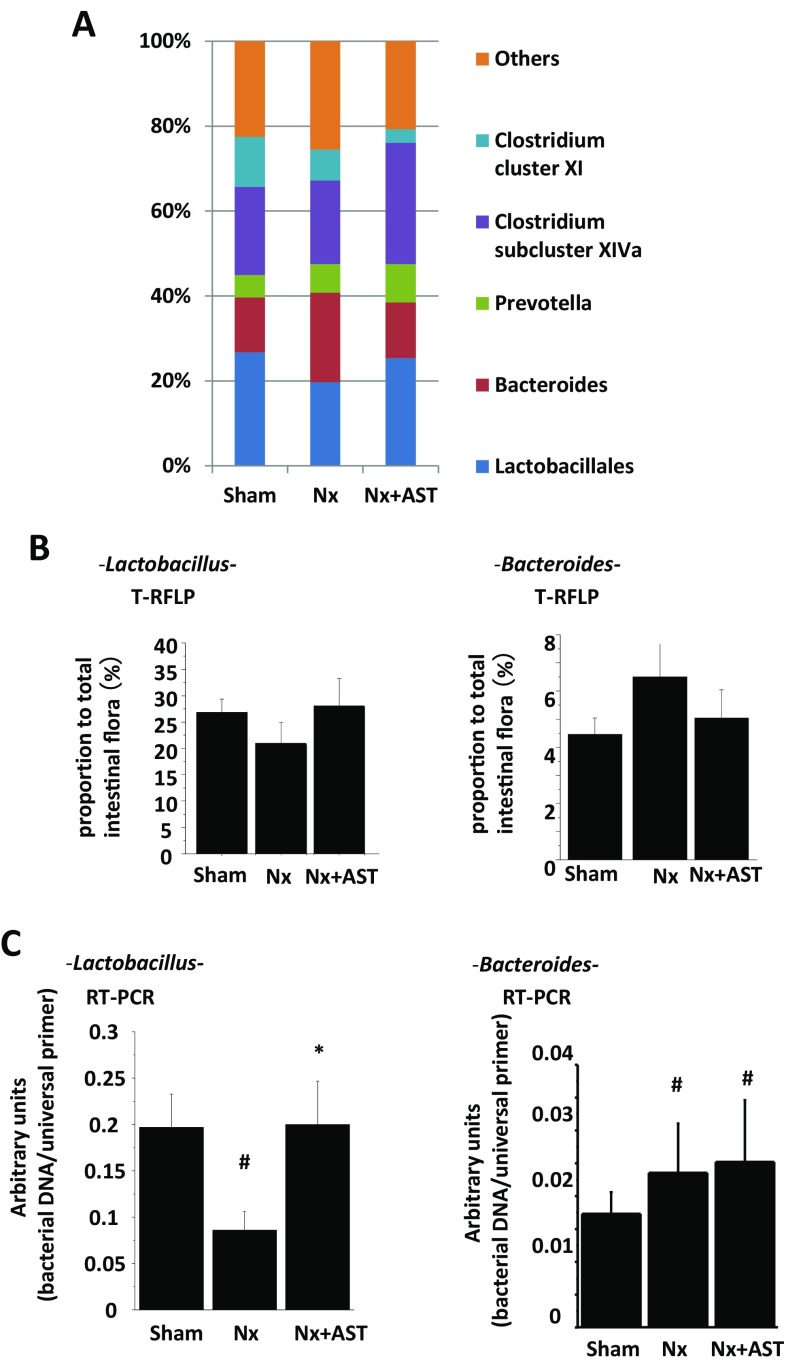
The effects of AST on microbiota population in nephrectomized rats. Spontaneously hypertensive rats underwent sham nephrectomy (Sham) or 5/6 nephrectomy (Nx) or Nx followed by AST treatment (Nx + AST). **a** Relative abundance of microbiota based on the average number of each subfamily was measured by T-RFLP analysis. **b** Populations of *Lactobacillus* species (left panel) and *Bacteroides* species (right panel) in the colons were examined by T-RFLP. **c** Populations of *Lactobacillus* species (left panel) and *Bacteroides* species (right panel) in the colons were examined by PCR. ^#^*P* < 0.05 versus Sham; *P < 0.05 versus Nx Sham: *N* = 9, Nx: *N* = 8, Nx + AST: *N* = 8

By the absorption of indole produced in the intestine (Fig. 2d), AST also would change the intestinal environment including the population of microbiota. To exclude the possibility that the AST induced the alteration of gut microbiota, we compared gut microbiota from terminal ileum between rats with and without AST treatment by T-RFLP method. The results showed no difference between the two groups. Therefore, AST itself had a little influence on the gut microbiota. Then, we evaluated intestinal microbiota by T-RFLP method among SHR, Nx and Nx + AST (Fig. 4a). Among all kinds of microbiota examined, we found that *Lact* species was decreased in Nx and the number was increased in Nx + AST. Confirmatory PCR analysis revealed that the changes in number of *Lact* were consistent with the results of T-RFLP (Fig. 4b, c, left panel). Additionally, *Bacteroides* (*Bact*) species was increased in number in Nx, which was decreased by AST (Fig. 4b, right panel). However, confirmatory PCR analysis revealed that increase in *Bact* number in Nx was not altered in Nx + AST (Fig. 4c, right panel). Other species of microbiota did not exhibit significant changes with the renal damages.

### The effects of the combination treatment with AST and *Lact* on CKD

**Fig. 5:**
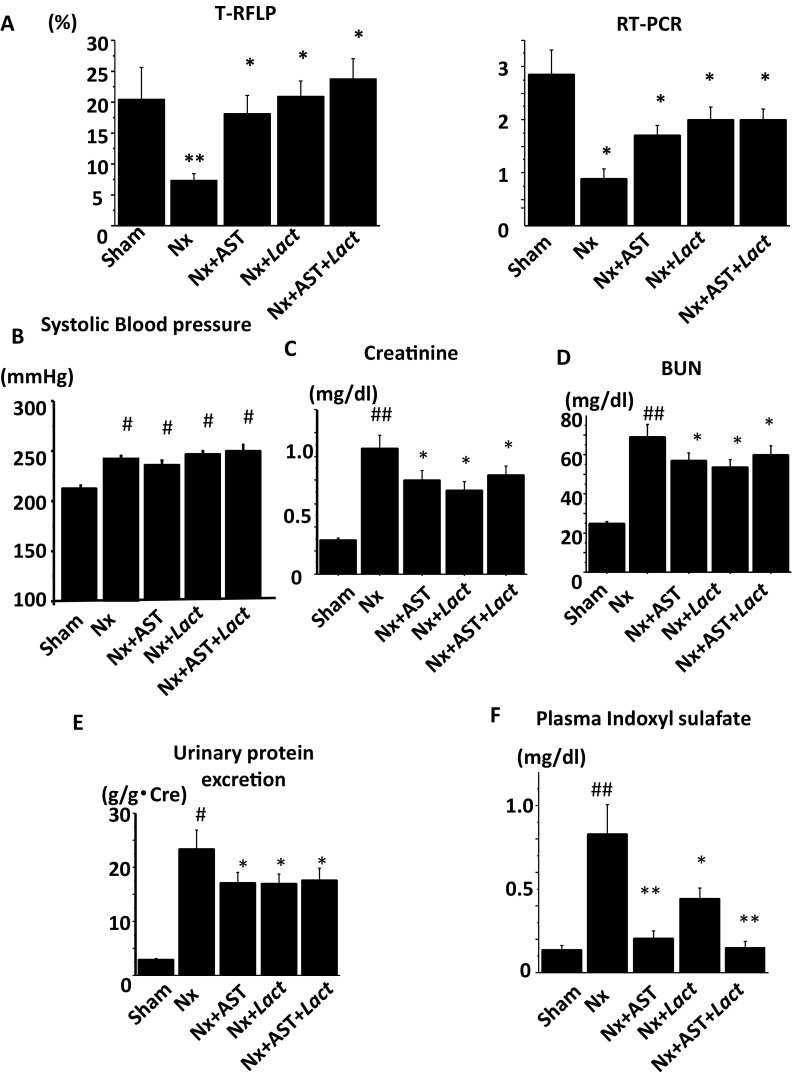
The effect of combination treatment with AST plus *Lact* in nephrectomized rats. Spontaneously hypertensive rats underwent sham nephrectomy (Sham), 5/6 nephrectomy (Nx), Nx plus AST treatment (Nx + AST), Nx plus *Lact* treatment (Nx + *Lact*), or Nx plus AST and *Lact* treatment (Nx + AST + *Lact*) as described in “Materials and methods”. **a** Populations of *Lact* species (left panel) in the colons of Sham, Nx, Nx + AST, Nx + *Lact*, and Nx + AST + *Lact* were examined by T-RFLP (left) and PCR (right). Measurements of systolic blood pressure (SBP, **b**), serum creatinine level (**c**), blood urea nitrogen level (BUN, **d**), daily urinary protein excretion (**e**), serum indoxyl sulfate (**f**) levels are compared among the groups. ^#^*P* < 0.05, ^##^*P* < 0.01 versus Sham; *P < 0.05, **P < 0.01 versus Nx. *N* = 8 per group

We previously showed the probiotic effects of *Lact* in CKD [13]. Therefore, we sought to evaluate the additional effect by *Lact* to AST treatment. We divided into five experimental groups: (1) Sham, (2) Nx, (3) Nx + AST, (4) Nx + *Lact*, and (5) Nx + AST + *Lact*. The population of *Lact* evaluated with T-RFLP and PCR revealed the increased number in *Lact* in Nx + AST, Nx + *Lact*, and Nx + AST + *Lact* as compared to that in Nx. However, there was no additional increase in *Lact* in Nx + AST + *Lact* as compared to those in Nx + AST or Nx + *Lact* (Fig. 5a). Although chow intakes were not different, body weight was decreased in Nx, which were ameliorated in Nx + AST, Nx + *Lact* and Nx + AST + *Lact* (Sham; 359.4 ± 10.1 g, Nx; 288.4 ± 28.0 g, Nx + AST; 327.1 ± 24.4 g, Nx + *Lact*; 324.1 ± 22.6 g, Nx + AST + *Lact*; 332.1 ± 23.2 g). SBP were not different among the groups (Fig. 5b). Serum creatinine levels were increased in Nx, which were not altered in Nx + AST, Nx + *Lact* and Nx + AST + *Lact* (Fig. 5c). The increase in BUN and in urinary protein excretion in Nx was also ameliorated in Nx + AST, Nx + *Lact* and Nx + AST + *Lact*, showing renoprotective effects by *Lact* or AST (Fig. 5d, e). Serum IS was increased in Nx, which was mitigated in Nx + AST, Nx + *Lact* and Nx + AST + *Lact* (Fig. 5f). However, regarding BUN, proteinuria, and IS, we failed to observe additional effects by combination treatments.

## Discussion

The present study demonstrated renoprotective effects through gut environment by AST in CKD rats. AST attenuated proteinuria, renal dysfunction, and glomerulosclerosis without affecting SBP.

Our previous paper showed that the renoprotective effects by *Lactobacillus* improve the decreased tight junction expressions through TLR2 in CKD rats. We explored the mechanisms for these effects by AST and found that AST ameliorated the decreased gut *Lactobacillus* and the decreased expression of tight junction in Nx through TLR2 and TLR4 pathway which was not observed in the single treatment with *Lactobacillus*. We also found the reduction of serum uremic toxins, which might contribute to the amelioration of renal damages. These systemic and renal favorable effects by *Lactobacillus* in combination resulted in the mitigation of renal tissue damages. We also demonstrated that there exist the upper limits of *Lactobacillus* by the combination experiment with AST and *Lactobacillus*.

AST is believed to reduce the serum levels of uremic toxins by capturing its precursors in the intestine and blocking the entry of these substances. In the present study, we have shown the increase in fecal excretion of indole, *p*-cresol, and phenol by AST and the reduction in serum IS, PCS and IAA. In addition, we proposed that the amelioration of intestinal barrier disruption contributed to the blockade of the entry of these toxic molecules. We showed the decrease in the expressions of intestinal tight junction in Nx, which were ameliorated by AST as was already demonstrated previously [11]. One of the important mechanisms for the restoration of tight junctions is the deprivation of uremic toxin such as indole in the intestinal lumen. As we previously reported that in vitro studies, indole downregulates the expressions of tight junctions with a concentration-dependent manner [13]. Recently, some papers focused on the protective effect of indole on the intestinal barrier. The difference among the papers might be caused by the models (the model of colitis or the model or CKD) and the amount of uremic toxins. However, the CKD condition, in which numerous and abundant uremic toxins accumulate in the intestine, at least, provokes the decrease of tight junction by the concentration.

Uremic toxins have been shown to activate aryl hydrocarbon receptor (AhR) [24–28] or pregnane X receptor (PXR) and the activation of PXR by indole derivatives regulated intestinal barrier expression through TLR4 [29]. TLR4 can be activated by circulating LPS derived from bacteria in the intestine [30]. Though LPS in the intestine is not absorbed by AST, the absorption of uremic toxins which reduced the activation of AhR or PXR leads to the decreased expression of TLR4 (Fig. 3b). These effects cumulated in the elevation of the intestinal barrier (Fig. 3a) and the decrease in the systemic inflammation, contributing to the slow progression of CKD.

As another plausible mechanism for the amelioration of intestinal barrier structure, we focused on the changes in microbiota by AST. In uremic conditions, microbes, such as *Clostridiaceae, Enterobacteriaceae* and *Verrucomicrobiacea*, which produce indole and p-cresol increased, whereas butyrate-producing microbes including *Lact* and *Prevotellae* decreased as compared with those of healthy subjects [31]. Our analysis showed the increase in *Bact* known as a species of indole producing microbe in Nx. We also demonstrated the restoration of *Lact* by AST. We previously demonstrated that the administration with *Lact* also reversed the tight junction disruption through TLR2 pathway in vivo and in vitro studies [13]. The increase of *Lact* by AST is also related to the upregulation of tight junction through TLR2 pathway since *Lact* is considered to be one of the key regulators to maintain and form the tight junction protein in the gut.

However, the causes why gut microbiota has changed by the AST treatment are still unknown. Recently, Mishima et al. reported that the ClC-2 chloride channel activator lubiprostone in CKD models mice improves the gut environment [32]. In the paper, they suggested that the retention of uremic toxins, intestinal ischemia, intestinal transit time prolonged by constipation, decreased intestinal fluid secretion, and malnutrition of gut lining with evident atrophy can provoke the shift of gut microbiota. In our case, there are two main causes for the improvement. One cause can be the decrease in the retention of uremic toxins by AST. The other cause can be the improvement from the intestinal ischemia by the improved expression of mucin-2 (the protective layer above the intestinal barrier). As *Lactobacillus* was shown to adhere to the mucin layer and survive as an intestinal microbiota. Therefore, AST attenuates the gut microbiota including the elevation of *Lactobacillus*.

Moreover, one of the main mechanisms for the renal protective effects by AST can be its anti-inflammatory effects through the reduction of serum IS. It was reported that Nx exhibited low-grade inflammation, as evidenced by increased level of serum IL-6. These effects might result in the impairment of structure and function of the kidney [33]. It is reported that IS locally induces reactive oxygen species (ROS), which activate the nuclear factor-kappaB (NF-kB) pathway and trigger both oxidative stress, pro-inflammatory cytokine production and renal tissue damages [34, 35]. Therefore, reduction of IS by AST teleologically mitigates renal damages. Moreover, we demonstrated the reduction in systemic inflammation by AST treatment. The mitigation of tight junction proteins and the restoration of intestinal barrier function would result in the blockade of LPS entry from the intestine and subdued inflammatory state in CKD.

In the previous report, the supplementation of *Lact* improved the renal damage and systemic inflammation [13]. The present data showed that AST also restored the renal impairment and systemic circulation of uremic toxins and systemic inflammation. Therefore, we hypothesized that the combination therapy with the *Lact* and AST could retain the additive effect. However, this therapy failed to show further effects. Of note, the fecal concentration of *Lact* did not show the significant change between Nx + AST and Nx + AST + *Lact* though the elevation of *Lact* concentration in Nx + AST + *Lact* is predicted. Thus, the population of *Lact* did not increase even after the addition of *Lact* orally in Nx + AST. This result indicated the upper limit of *Lact* residing in the intestinal environment. Similarly, in the previous report, treatment with *Lact* did not show dose-dependent effects. Petschow et al. demonstrated that although they administered three different concentrations of *Lact* (10^8^, 10^9^, 10^10^ cfu/day) the number of fecal *Lact* did not differ among three groups [36]. Therefore, our data implied that effects by AST including the improvement of the intestinal environment and the alteration of microbiota considered to have the upper limit. This limitation was considered to be defined by the population of *Lact*. This also suggests that the effects AST are partly dependent on the alteration of microbiome and are not solely dependent on the serum uremic toxin levels. This mechanism would explain why recent large scale clinical trial in EPIC study failed to show the favorable effects on renal function [8], while some small clinical trials in Japan have succeeded [9, 10], since the population of microbiota is to vary among the different races or different food consumptions.

As a limitation, we cannot evaluate all the gut microbiota quantitatively by T-RFLP. Though this method is less difficult and indicates the accurate trends, the resolution is not so high because different DNA fragments sometimes show the same bands because of the restriction enzyme. After T-RFLP methods give us the general trends, confirmatory real-time PCR would be better for more accurate data. Then, this paper mainly focuses on *Lactobacillus* and *Bacteroides*, which has some trends with renal function.

In conclusion, AST improved gut environment favorable to *Lact* which affected the tight junction expressions though TLR pathway with renal protective effects. Our data may help to establish more efficient strategy against CKD with the use of AST in clinical practice.
